# Novel 5′ Untranslated Region Directed Blockers of Iron-Regulatory Protein-1 Dependent Amyloid Precursor Protein Translation: Implications for Down Syndrome and Alzheimer's Disease

**DOI:** 10.1371/journal.pone.0065978

**Published:** 2013-07-31

**Authors:** Sanghamitra Bandyopadhyay, Catherine Cahill, Amelie Balleidier, Conan Huang, Debomoy K. Lahiri, Xudong Huang, Jack T. Rogers

**Affiliations:** 1 Neurochemistry Laboratory, Department of Psychiatry, Massachusetts General Hospital and Harvard Medical School, Charlestown, Massachusetts, United States of America; 2 Department of Pediatrics, Massachusetts General Hospital and Harvard Medical School, Charlestown, Massachusetts, United States of America; 3 Laboratory of Molecular Neurogenetics, Department of Psychiatry, Institute of Psychiatric Research, Indiana University School of Medicine, Indianapolis, Indiana, United States of America; Thomas Jefferson University, United States of America

## Abstract

We reported that iron influx drives the translational expression of the neuronal amyloid precursor protein (APP), which has a role in iron efflux. This is via a classic release of repressor interaction of APP mRNA with iron-regulatory protein-1 (IRP1) whereas IRP2 controls the mRNAs encoding the L- and H-subunits of the iron storage protein, ferritin. Here, we identified thirteen potent APP translation blockers that acted selectively towards the uniquely configured iron-responsive element (IRE) RNA stem loop in the 5′ untranslated region (UTR) of APP mRNA. These agents were 10-fold less inhibitory of 5′UTR sequences of the related prion protein (PrP) mRNA. Western blotting confirmed that the ‘ninth’ small molecule in the series selectively reduced neural APP production in SH-SY5Y cells at picomolar concentrations without affecting viability or the expression of α-synuclein and ferritin. APP blocker-9 (JTR-009), a benzimidazole, reduced the production of toxic Aβ in SH-SY5Y neuronal cells to a greater extent than other well tolerated APP 5′UTR-directed translation blockers, including posiphen, that were shown to limit amyloid burden in mouse models of Alzheimer's disease (AD). RNA binding assays demonstrated that JTR-009 operated by preventing IRP1 from binding to the IRE in APP mRNA, while maintaining IRP1 interaction with the H-ferritin IRE RNA stem loop. Thus, JTR-009 constitutively repressed translation driven by APP 5′UTR sequences. Calcein staining showed that JTR-009 did not indirectly change iron uptake in neuronal cells suggesting a direct interaction with the APP 5′UTR. These studies provide key data to develop small molecules that selectively reduce neural APP and Aβ production at 10-fold lower concentrations than related previously characterized translation blockers. Our data evidenced a novel therapeutic strategy of potential impact for people with trisomy of the APP gene on chromosome 21, which is a phenotype long associated with Down syndrome (DS) that can also cause familial Alzheimer's disease.

## Introduction

Many RNA-binding protein interactions are closely associated with neurological and psychiatric disease processes such as amyotrophic lateral sclerosis (ALS) [1] and autism [2]. In this report, we sought proof that the use of APP translation blockers can reduce amyloid expression pertinent to providing therapy for individuals afflicted with Alzheimer's disease (AD) and Down syndrome (DS).

Increased levels of the metals iron, copper, zinc in the brain are associated with increased risk to accelerate the course of AD [3]. To safely store excess iron, canonical iron-responsive elements (IREs) are the 5′UTR-specific RNA stem loops that control translation of L- and H-ferritin mRNAs (iron storage) so that the L- and H chains can assemble into this iron storage multimer. The iron-regulatory proteins (IRP1 (90 kDa) and IRP2 (105 kDa)) are the two known RNA-binding proteins that are key gatekeepers for cellular iron homeostasis because of their inducible interaction with IREs to control ferritin mRNA translation and transferrin receptor (TfR) mRNA stability (iron uptake) [4].

Consistent with our report that APP is an iron export ferroxidase [5], RNAi knockout studies showed that IRP1 binds strongly to 5′UTR sequences in the APP transcript to repress expression of the precursor [6]. In fact, the APP mRNA encodes an active IRE that binds with a different RNA-binding specificity to IRP1 relative to the IRE of ferritin mRNA (which interacts with IRP1 & IRP2) [6]. Thus the APP 5′UTR is a unique, highly specific drug target to identify APP (and Aβ) repressors. This model is consistent with a recent report that IRP1 outcompetes IRP2 in regulating cellular iron homeostasis in response to nitric oxide [7].

The concept of repressing APP translation as a therapeutic strategy in DS and AD was proven as a novel anti-amyloid strategy as exemplified by our use of the APP 5′UTR-directed FDA drug N-acetyl-cysteine (NAC) in the TgCRND8 APP(Swe) mouse model of AD [8]. An additional benefit of limiting the APP levels may be to restore perturbations to iron homeostasis during DS since APP is over-expressed by one third on the DS trisomy chromosome 21 [9]. Increased APP may well alter brain iron homeostasis based on its capacity to bind ferroportin and export iron [5]. In this regard, mice that are trisomic for chromosome 16, the orthologue of human chromosome 21, over-express APP and are genetically shown to develop the DS phenotype because of a triplicated expression of the APP gene [9], [10]. The progression of familial Alzheimer's disease (FAD) can be the result of a genetically inherited over-expression of the APP gene or by somatically induced non-disjunction events that cause APP to be over-expressed [11], [12], [13].

Thus, in addition to the altered processing of APP and other risk factors (e.g., inflammation, metal-catalyzed oxidative stress [3], [14], [15], [16], and the increased levels of apolipoprotein-E [17], [18] and α-1 anti-chymotrypsin (ACT) [19]), simple elevation of APP levels is a sufficient genetic cause of DS and AD [12], [20]. This report centers on our RNA targeting strategy as a starting point to develop drugs that can limit APP expression by a novel therapeutic mechanism for offsetting APP mRNA translation rates and reducing severe amyloidosis during the progression of DS and AD.

The proven *in vivo* efficacy of APP 5′UTR-acting FDA drugs, including NAC and the APP translation blocker posiphen, encouraged us to pursue a high-throughput screening campaign against APP 5′UTR in search of potent and selective APP translation blockers [21].

We identified and characterized a novel APP 5′UTR-specific translation blocker of neuronal APP and Aβ that operates at nanomolar concentrations while maintaining β-actin expression and cell viability [9]. JTR-009 is a benzimidazole that was found to reduce intracellular APP and toxic Aβ production in both SH-SY5Y neural cell lines and primary mouse neurons. Here we have shed light on the mechanism of action of JTR-009, which is consistent with the drug intercalating into RNA sequences folded from the APP 5′UTR and irreversibly replacing IRP1 as the repressor of APP translation. These findings supported our pharmacological goal, as described in this report, to reduce APP expression with therapeutic implications particularly for DS and AD.

## Materials and Methods

### 1. Antibodies

Rabbit anti-human IRP1 antibody (Alpha Diagnostics International, San Antonio, TX) and anti-IRP1 (kind gift from Dr. Sharon Cooperman and Dr. Tracey Rouault, National Institutes of Health) each generated the same results in the assays shown; mouse anti-human IRP2 (Santa Cruz Biotechnology, Santa Cruz, CA) detected the H-ferritin IRE-IRP2 interaction, and a second antibody to IRP2 was also utilized to confirm the selectivity of IRP2 binding as detected with the Santa Cruz Biotechnology antibody. Anti-β-actin, anti-α-tubulin, rabbit anti-APP C-terminal antibody (A8717) were from (Sigma, St. Louis, MO), and the APP N-terminal antibody (22C11) was from Chemicon (Temecula, CA).

### 2. Cell Culture and Preparation of Lysates

Human SH-SY5Y neuroblastoma cells were cultured in DMEM supplemented with 10% FBS (Invitrogen, Carlsbad, CA) and penicillin/streptomycin (Bio-Whittaker, Walkersville, MD). Cells were exposed to JTR-009 (0–100 µM, Calbiochem) and iron (50 µM, National Institute of Standards and Technology (NIST), Gaithersburg, MD), provided to cells as ferrous ammonium sulfate. Cytoplasmic protein lysates were prepared by homogenizing the cells in ribonucleoprotein immunoprecipitation buffer (25 mM Tris, pH 7.4, 1%Nonidet P-40, 0.5% sodium deoxycholate, 15 mM NaCl, protease inhibitors, RNase inhibitor, and 10 µM DTT). For preparation of conditioned medium for Aβ and LDH measurements, cells were treated for 48–72 hours with each compound as described in the legends. 1 mL was used for total Aβ determination by ELISA as described by Biosource International. according to maufacturers conditions (see Ref [8]).

Primary cortical neurons from wild type mice and from the PAC-Tg(*SNCA*(wt) human *SNCA* genomic mice [22] were cultured as outlined by the method of Ray et al., 2009 [23]. We recovered the embryonic (E15-18) pups after sacrificing pregnant females, separated out the brain, and removed the meninges and blood vessels. We then dissected out the cortices and placed them in separate Eppendorf tubes containing 500 µL of HBSS without Ca^+2^/Mg^+2^ salts supplemented with 1 mM sodium pyruvate and 10 mM HEPES, pH 7.4. On ice, individual cells were isolated by titrating 10 times using a glass pasture pipette with the tip barely fire polished. We adjusted the volume to 1.5 mL by adding 1 mL of HBSS with Ca^+2^/Mg^+2^ salts+Na. pyruvate+HEPES, restoring the divalent cations by adding HBSS so that the non-dispersed tissue could settle for 5 min, on ice. In the tissue culture laminar hood, we transferred the supernatant into a new 15 mL tube and centrifuged for 1 min at 900 rpm, 4°C. We gently re-suspended the pellet in 2 mL of HBSS with Ca^+2^/Mg^+2^ salts+Na pyruvate+HEPES and took an aliquot for counting (2 mL for approx 5 embryos). We then plated ∼1×10^5^ cells/well of a 24 well or 2×10^5^/in 12 well plates. Each set of plates was coated with poly D-lysine containing poly L-lysine coverslips for micro immuncytochemical confirmation of neuronal integrity.

### 3. Methodology of Molecular Screens

We screened the 110,000 compounds of the molecular library of LDDN at Harvard to identify novel and more potent APP 5′UTR-directed inhibitors [21]. The LDDN library had already yielded small molecules that inhibit mesangial cell proliferation [24], following three-dimensional pharmacophore modeling and screening. A second Molecular Libraries Screening Centers Network HTS was conducted at the Columbia University Genome Center to generate hits as listed on PUBCHEM (AID: 1285), from which our dose-response assays identified 50 lead APP 5′UTR-directed luciferase reporter inhibitors. Two classes of APP 5′UTR-directed translation blockers from the second screen exhibited a potent IC_50_ in the 10^−8^ M range. We pooled a shortlist of the thirteen most selective APP 5′UTR inhibitors from both screens. These thirteen leads were tricyclic aromatic compounds that included two major classes of hits: compounds with a benzimidazole backbone, i.e. APP blockers -2, -7, and -9 (JTR-009) and compounds with a benzothiazole backbone, i.e. APP blockers -8 and -13. The compounds with a benzothiazole backbone were also identified to be similar to PFTα, another benzothiazole, and P53 inhibitor, by showing protection against oxidative injuries in synaptosomes from wild-type mice and preserving presynaptic terminals in cultured hippocampal neurons exposed to etoposide [25] [26]. The anti-APP 5′UTR efficacy of the 13 top inhibitors was directly compared with their anti-APP efficacy by Western blotting of lysates prepared from SH-SY5Y cells.

### 4. Western Blotting

After cells were exposed to increasing concentrations of the compounds as outlined in each figure legend, cytoplasmic protein lysates were prepared by homogenizing the cells in midRIPA buffer (25 mM Tris pH 7.4, 1% NP40, 0.5% sodium deoxycholate, 15 mM NaCl, protease inhibitors, RNase inhibitor and 10 µM DTT). Western blotting for APP was performed using the N-terminal 22C11 antibody (Millipore, inc) and the A8717 C-terminal specific APP antibody (Sigma, inc), while αsyn was detected using mouse monoclonal anti-αsyn (BD Transduction Laboratories) and anti-β-actin was from Chemicon. The blots were developed using chemiluminescence (PIERCE, Rockford, IL) and visualized with a Phosphoimager (BioRad, Hercules, CA). The bands were quantified using QuantityOne® software (BioRad).

### 5. RNA Quantitation

We conducted qRT-PCR to measure the capacity of JTR-009 to change steady state levels of APP mRNA levels, as was previously described (see Ref [6]). Desferrioxamine treatment for 48 h was used for a positive control to assess changes to the steady state levels of both APP mRNA and transferrin receptor mRNAs. Primers for β-actin were employed as a control for an mRNA previously shown to be unchanged by desferrioxamine and other inducers [6]. Experiments were carried out on the ABI Prism 7000 sequence detection system (Applied Biosystems). Total RNA was isolated using TRIzol reagent (Sigma) according to the manufacturer's instructions. cDNA was synthesized with SuperScript III first-strand qPCR supermix (Invitrogen) according to the manufacturer's instructions. The primers to β-actin, TfR1) were designed and ordered from Invitrogen. The APP primer set was purchased from Qiagen and has been benchmarked on several reports for accurate measurement of APP mRNA levels.

### 6. Transfections and Luciferase Reporter Assays for Counterscreens

APP 5′UTR-Luciferase inhibitor compounds obtained from the preliminary HTS of pIRES-APP-5′UTR transfectants were picked, and the dose-response assays were conducted at 0.1, 1.0, and 5.0 µM (based on the exact molecular weights of the compounds). For the purpose of counter-screening, pIRES-PrP-5′UTR)-transfected SH-SY5Y cells were plated in 384-well black plates, and the identified compound hits that were not cytotoxic were manually added to the cells. Each hit was added in 5 wells, and this was repeated twice on 2 different days. There was a positive control and negative control column of cells as previously described [21]. The inhibition of luciferase was calculated, and the average of the values obtained was considered (see data shown in Table 1).

**Table 1:** IC_50_ (nM) for APP 5′UTR blockers in pIRES-APP-5′UTR transfectants (top row) and pIRES-PrP-5′UTR transfectants (bottom row).

Agent (nM)	1	2	3	4	5	6	7	8	9	10	11	12	13
IC_50_	700	800	750	**80**	5000	1000	500	80	**400**	**900**	**3,000**	3000	**100**
IC_50_	700	5000	750	**400**	5000	1000	1000	80	**4000**	**2000**	**10,000**	7000	**500**

Values were calculated from inhibiton curves in 384-plate assays to reduce 5′UTR driven luciferase expression [21].

### 7. Biotinylated RNA Pulldown Assay

Biotinylated RNA oligonucleotides: H-ferritin IRE (biotin-5′-GGG UUU CCU GCU UCA ACAGUG CUU GGA CGG AAC CCG G-3′) and APP IRE (5′-biotin-GC GGU GGC GGC GCG GGC AGA GCA AGG ACG CGG CGG AU-3′) were purchased from Invitrogen. Cell lysates (100 µg) were incubated with100 nM biotinylated oligonucleotide for each of the IREs, for 3 h at room temperature. Paramagnetic streptavidin-conjugated Dynabeads (Invitrogen) were washed with ribonucleoprotein immunoprecipitation buffer, added into lysates to bind IRP(1/2)-biotinylated-RNA complexes, and incubated for 1 hour at room temperature. After five washes, the proteins that bound to the beads were analyzed by Western blotting for IRP1, IRP2, and biotin. The blots were developed with chemiluminescence (Pierce) and visualized with a 4000 MP VersaDoc™ Imaging System (Bio-Rad). The IREs-bound IRPs were quantified by Quantity One® software (Bio-Rad).

### 8. Calcein Assay

Cells were loaded with calcein after incubation with 0.1 µM of Calcein-AM for 10 min in 0.15 M NaCl-20 mM HEPES buffer, pH 7.4, with 0.1% BSA at 37°C, an action followed by extensive washing with NaCl-HEPES buffer to remove extracellular bound calcein. The cells were aliquoted at 5×10^4^–1×10^5^ cells/well in 96-well plates containing test compounds at 10 µM and incubated for 30 min in a humidified 37°C incubator with 5% CO_2_ before baseline fluorescence was obtained at 485/520 nm (excitation/emission) with 0.1% DMSO, as the vehicle control, and DTPA as a strong iron chelator control to block all iron uptake. Using Using a SpectraMax M5^e^ plate reader and SoftMax Pro software (Molecular Devices, Sunnyvale, CA), the fluorescence was then measured 30 min after the addition of 10 µM ferrous ammonium sulfate in 500 µM ascorbic acid (AA). The percentage of fluorescence quench was calculated relative to 200 µM DTPA, which was added as a blocking control, and DMSO as a vehicle control, as follows:
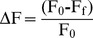
(1)where Δ F is the change in fluorescence, or fluorescence quench observed in any well. F_0_ represents the fluorescence after 30 min of the compound, and F_f_ represents the fluorescence 30 min after addition of Fe. These results were normalized to the blocking and vehicle controls as follows:
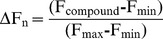
(2)where ΔF_n_ is the normalized quench observed after addition of iron. F_compound_ is the Δ F observed with the compound, F_min_ is the average Δ F of the DMSO control, and F_max_ is the average ΔF of the DTPA control.

With this normalization, 100% indicates that the test compound is as potent as DTPA in blocking iron-induced quenching, and 0% indicates no inhibition of iron quenching by the test compound or the same quench as observed with the DMSO vehicle control. Compounds with ΔF_n_ between 0% and 100% are defined as inhibitors of iron uptake. Negative values for ΔF represents compounds that facilitate iron uptake into cells. Our criteria for active compounds to be further investigated were arbitrarily set as ΔF_n_ = 50–100% quenching for iron uptake inhibitors and <−50% quenching for iron uptake facilitators.

### 9. ELISA Measurement of Secreted Aβ levels and Lactase Dehydrogenase (LDH)

After reaching 80% confluence, SH-SY5Y cells were 1∶3 split onto two 12-well plates. After allowing the cells to settle for 24 hours, the medium was switched to a 1% FBS DMEM (Dulbecco's modified essential medium supplemented with 1% FBS and penicillin/streptomycin).

#### Aβ Assays

Total Aβ amyloid levels were assessed as we previously described (8) and Aβ-42 levels were measured by use of ELISA according to manufacturers instructions (Covance Chemiluminescent BetaMark x-42 ELISA).

#### LDH assay

The 1% FBS medium was recommended by the LDH Cytotoxicity Kit (Cayman Chemical, Ann Harbor, MI) to reduce interference as FBS also contains LDH. Cells were exposed to 10-fold increases in concentrations of JTR-009 reconstituted in 1× PBS (0.1 nM-100 µM) compared to PBS as a control for 48 hours. Thus, eight wells on each 12-well were treated for 48-hour after which time 100 µL of supernatant was extracted from each of the treated wells and transferred to a 96-well plate. A LDH standard from the kit was also added to the plate. Using the reaction mixture in the kit, LDH absorbance values were obtained with a SpectraMax M5e plate reader and SoftMax Pro software ((Molecular Devices, Sunnyvale, CA)).

### 10. MTS Assay for Neuronal Viability

Cell viability was determined using MTT (thiazolyl blue tetrazolium) viability assays. Cells were grown in 96 well plates and treated as indicated above. After treatment, they were incubated with 20 µL of 5 mg MTT (Sigma)/1 mL PBS solution for 3.5 hours. The media was aspirated from the cells and 150 µL of solvent (0.1% Nondet P-40, 4 mM HCl in isopropanol) was added to each well and the plate shaken for 15 minutes. The absorbance was then read at 590 nm using a SpectraMax M5e plate reader and SoftMax Pro software (Molecular Devices, Sunnyvale, CA).

## Results

### A: Selectivity of APP 5′UTR translation blockers from PrP 5′UTR-based counter-screen

**Figure 1:**
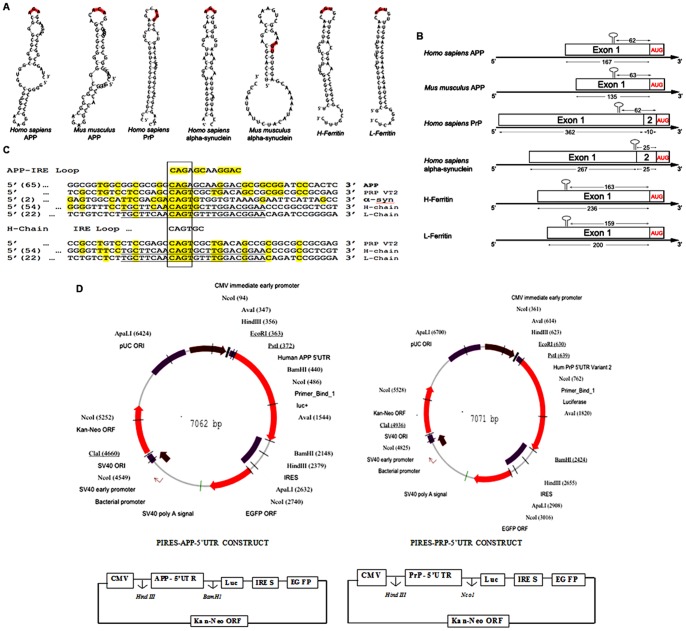
Alignment of human and mouse APP 5′UTRs with human PrP 5′UTR sequences relative to the L- and H-ferritin Iron-responsive elements (IREs). **Panel A**: The human and mouse APP 5′UTR specific IRE-like RNA stem loops, the human PrP 5′UTR, and the human and mouse *SNCA* specific IRE –like stem loops each aligned adjacent to the ferritin L- and H IRE RNA stem loops. Shown, the L- and H-mRNAs encode canonical IRE RNA stem loops whereas the APP IRE in non canonical although fully iron responsive [6]. The α-synuclein IRE (*SNCA* IRE) represents a non canonical IRE traversing the central splice junction of exon-1 and exon-2 (the CAGUGN loop/splice site sequences) of *SNCA* mRNA [49]. Typical IRE stem loops fold to exhibit an apical AGU pseudotriloop which is depicted in red lettering at the apex of the H-ferritin and *SNCA* IREs [28] relative to an analogous AGA from the IRE–like stem loop encoded by APP mRNA [6]. **Panel B**: Maps of the 5′UTRs encoding by the human and mouse APP mRNAs, PrP mRNA, *SNCA* mRNA, and the mRNAs for L- and H-ferritin (IRE stem loops are displayed as lollipops). **Panel C**: Relative alignment of the sequences that encode the 5′UTR specific IRE-like stem loops in human APP mRNA, PrP mRNA, *SNCA* mRNA, and the L- and H-ferritin mRNAs. **Panel D**: Screen and counter-screening Constructs [**21**] **:** The human APP 5′UTR cassette was subcloned in front of the luciferase reporter gene in the dicistronic pCD(APP) reporter construct. The same-sized and related PrP 5′UTR was subcloned in an identical format into the pCD(PrP) reporter construct for the purpose of counter-screening, as described in the materials and methods section.

In Figure 1, Panel A shows the specific RNA stem-loops encoded by the 5′UTRs of several neurodegenerative disease transcripts, specifically those for APP, PrP, and α-synuclein (*SNCA*). Each mRNA encodes uniquely configured variations of an IRE RNA stem loop that potentially bind to the IRP translational repressors in their 5′UTRs. The prion PrP 5′UTR was chosen as a stringent counter control for ensuring that APP 5′UTR directed compounds would be sufficiently specific not to inhibit luciferase reporter gene expression in matched PrP 5′UTR-driven transfectants. Panels B and C of Figure 1 shows the maps and alignments of the 5′UTRs encoding IRE stem loops in the neurodegenerative transcripts for APP and αsyn relative to the canonical ferritin L- and H- chain IRE stem loops [21]. Figure 1C presents that this homology extends to that of the PrP 5′UTR, which encodes an IRE-like sequence, although diverged from the proven APP IRE (NCBI, Clustal software, [27]). Alignments elucidated a 56% similarity between this region of the 5′UTR of PrP mRNA (splice variant-2) and APP IRE sequences. This homology is centered around the CAGUGN loop domain of the canonical ferritin IRE and the projected IRP1 binding AGU/AGA tri-loops that were shown to be key for IRP1 and IRP2 binding and translation repression [6], [28]. The PrP(Vt2) 5′UTR was therefore deemed a stringent screening control to ensure specificity of our APP 5′UTR-directed translation blockers. Figure 1D shows the complete coordinates of the screening constructs, pIRES-APP-5′UTR and pIRES-PrP-5′UTR, which were matched for insertion of equal length 5′UTRS for screen/counter-screen comparisons in transfection based assays (Bandyopadhyay et al., 2006).

We conducted a screening campaign of library of 110,000 compounds with the stable transfected SH-SY5Y cells expressing the constructs shown in figure 1D. To identify APP 5′UTR-specific translation blockers from LDDN Harvard (see ref [21]) and from the Columbia University Genome center, we then counter-screened against the PrP 5′UTR and shortlisted thirteen potent inhibitors to be further characterized. In Figure 1D, we employed the listed constructs to conduct these transfection based assays to ensure that the 13 APP specific leads were not also PrP inhibitors. Table 1 lists the IC_50_ of each of these inhibitors with respect to their dose-responsive capacity to reduce APP 5′UTR-driven luciferase expression relative to their IC_50_ values against PrP 5′UTR expression. In Table 1, our calculations demonstrated a satisfactory >5-fold difference in IC_50_ values for APP blockers JTR-004, JTR-009 and JTR-0013 and 3-fold difference for JTR-0010 and JTR-0011 (shown in bold lettering).

Several APP 5′UTR blockers exhibited closer-than-expected differences in IC_50_ values when counter-assayed in dose response experiments against the PrP (vt-2) 5′UTR. To explain this finding, we noted that these constructs and cell lines encoded 100 nucleotide RNA targets with unexpectedly closeness of 56% sequence identity by gap alignment between the 5′UTR of the APP and the PrP transcripts, as described previously [21]. This finding is consistent with recent reports that PrP is an iron exporter similar to APP (ferroxidase-II), underlying their newly found functional equivalence in addition to each being pathogenic proteins [5], [29]. For this reason, of the thirteen confirmed leads (designated JTR-001 through JTR-0013), several may indeed provide a new class of agents that limit expression of both APP and PrP. All thirteen leads increased cell viability in SH-SY5Y cells as measured by the MTT assay (see MTS assays for JTR-005 and JTR-009 (next section).

Of the remaining leads that exhibited low toxicity and high selectivity towards APP 5′UTR sequences, three (JTR-002, JTR-007, JTR-009) harbored planar tricyclic benzimidazole backbones, and two (JTR-008, JTR-0013) were benzothiazoles. JTR-009 was selected for further studies as a result of two independent transfection-based determinations, which showed that this benzimidazole 10-fold more potently inhibited APP 5′UTR driven translation relative to the PrP 5′UTR (Table 1). The anti-stroke agent, pifithrin-alpha (PFTα), was a closely related benzothiazole to JTR-0013, and was previously found to be a selective APP translation inhibitor in our assays. PFTα was already shown to be an *in vivo* acting inhibitor of tumor suppressor protein p53 [25] whereas we noted other benzothiazole leads were similar to the Pittsburgh compound B (PiB) [30], [31].

**Figure 2:**
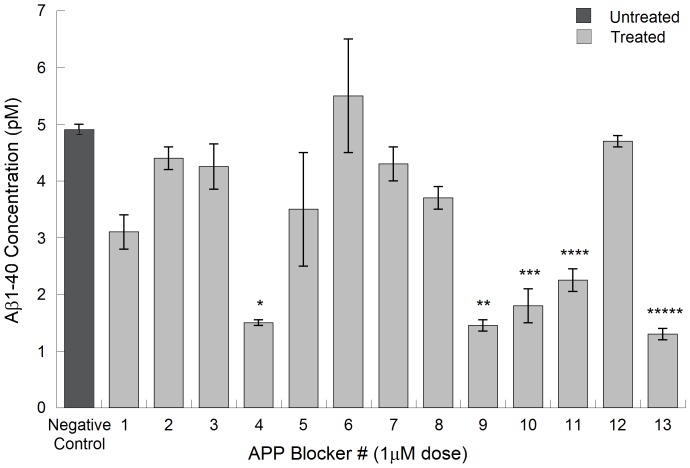
Relative capacity of thirteen APP 5′UTR translation blockers to reduce Aβ levels in the conditioned medium of SH-SY5Y cells. Following 48 h treatment (1 µM) for each inhibitor, the histogram shows reduction of total Aβ levels confirmed after averaging five independent samplings from the following:- JTR-009 treated<control, p<0.01). Total Aβ levels were also documented for the APP blockers JTR-004, JTR-10, JTR-0011 JTR-0013 (N = 5). Data are means ± SEM, N = 5, * = p<0.01, ** = p<0.01, *** = p<0.0013, p<0.01, **** p<0.011, *****, where each treatment was analyzed by ANOVA+Dunnett's post hoc test compared to untreated samples. JTR-009 was the ninth and JTR-005 was fifth in the series of 13 APP translation blockers.

We next measured the relative extent to which the top thirteen APP 5′UTR blockers reduced Aβ secretion from SH-SY5Y cells after 48 hours of 1 µM treatment. Five drugs showed significant reductions in levels of total Aβ peptide (JTR-004 (3-fold inhibition), JTR-009 (3-fold inhibition), JTR-0010 (2-fold inhibition), JTR-0011 (2-fold inhibition) and JTR-0013 (3-fold inhibition). With the exception of JTR-006 and -0012, each of the other APP 5′UTR directed inhibitors (1 µM dose) modestly reduced Aβ levels as measured by use of an ELISA (Biosource Int.) (See Figure 2), a finding that confirmed which APP translation blockers reduced levels of cellular APP template sufficiently enough to reduce Aβ peptide output from SH-SY5Y cells.

**Figure 3:**
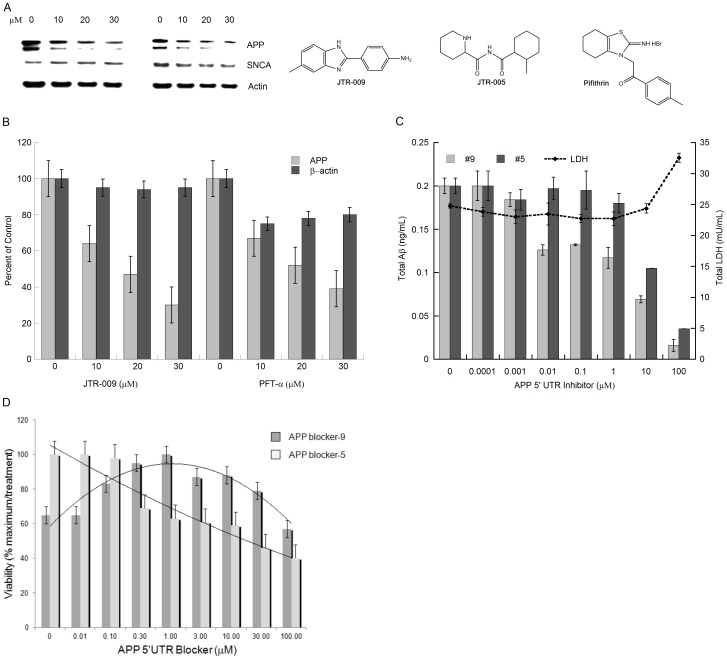
The effect of JTR-009 to reduce the steady state levels of APP in SH-SY5Y cells with a high degree of selectivity in the absence of changes to the levels of β-actin and α-synuclein (*SNCA*). **Panels A and B**: Dose-responsive (0, 10 µM, 20 µM, 30 µM) treatment of SH-SY5Y cells for 48 h to measure the capacity of JTR-009 and PFT-α to limit APP expression relative to β-actin and *SNCA* levels. The representative western blot experiment in Panel A contributed to densitometry for the histogram shown in Panel B (N = 3). Right Panel: Chemical structure of JTR-009, 4-(5-methyl-1H-benzimidazol-2yl) aniline, compared to the anti-apoptotic stroke agent PFTα, (275 Da), a tricyclic benzothiazole. **Panel C**: Dose-responsive measurement of total amyloid Aβ levels in response to the APP 5′UTR inhibitors JTR-005 and JTR-009, measured by benchmarked ELISA in conditioned medium of 72-hour treated SH-SY5Y cells. Shown are the mean values for the reduction of levels of Aβ ± SEM (N = 4) after treatment of the cells with JTR-009 and JTR-005 at 0.01 µM (* = p<0.01), 0.1 µM (** = p<0.015), and 1 µM (*** = p<0.01) analyzed by ANOVA (N = 5). Dotted line: Representative LDH assay parallel to Aβ determination for SH-SY5Y cells treated for 72 h at concentrations up to 100 µM of JTR-009 (N = 4). **Panel D**: MTS assay for cellular mitochondrial viability after treatment of SH-SY5Y cells with JTR-005 and JTR-009 at the concentrations shown. Y axis: Percent of maximal viability ± SEM after treatment of the cells with JTR-009 and JTR-005 (N = 3)). Shown are the relative trend-lines for the dose-responsive viability of JTR-005 and JTR-009 compared to untreated cells (‘poly’ = non linear polynomial regression of the data).

Of these, JTR-009 has consistently provided maximal cell viability (MTT assays see Fig. 3D). Thus JTR-009, as a translation blocker of APP mRNA, was sufficiently specific towards APP 5′UTR sequences and was considered to be a bonafide anti-amyloid agent (inhibition of Aβ by JTR-009 was 3-fold, ANOVA: p = 0.0046, N = 5 by pair-wise comparison of groups). JTR-009 was advanced for further analysis of the mechanism of the APP 5′UTR as a regulatory domain for APP gene expression at the level of message translation and as a candidate for future analog-based drug development as an anti-APP and anti-Aβ blocker for potential DS and AD therapy.

### B. JTR-009: The most selective and potent of the thirteen top APP 5′UTR-directed translation blockers

Consistently, JTR-009 was a highly specific APP 5′UTR translation blocker of luciferase reporter gene expression and also of steady state levels of APP (see Table 1 and Figure 3). (Significantly, JTR-009 was an equally potent suppressor of Aβ peptide levels (Figures 2 and 3) [21]. Pifithrin (PFT-α), a well tolerated anti-apoptotic drug that has a benzothiazole structure similar to JTR-0013, was employed for the purpose of comparison with JTR-009. Therefore, as proven APP 5′UTR inhibitors, the benzimidazole JTR-009 and the benzothiazole pifithrin were compared for their relative capacities to limit APP expression in SH-SY5Y cells. In Figure 3A, a representative Western blotting experiment demonstrated that both JTR-009 and PFTα dose dependently reduced APP translation. In Panel B densitometry quantified from five separate experiments, including the one shown, demonstrated a 50% reduction of APP at 20 µM concentrations (48 h treatment) after standardization for β-actin. JTR-009 reduced APP levels to 30% of control levels at 30 µM, while maintaining both β-actin and α-synuclein (*SNCA*) levels (N = 5, p = 0.003). Several similar western blots experiments showed that JTR-009, but not PFTα, had sufficient specificity to limit APP while also maintaining β-actin and αsyn levels (48 h). We consistently found that PFTα, which has a benzothiazole backbone like JTR-0013, inhibited neural APP with the same potency as JTR-009 but was less specific since PFTα co-reduced αsyn levels (another IRE encoding mRNA [32] as well as β-actin (Figure 3A, B).

Dose-responsive comparisons of JTR-009 with another APP 5′UTR-screened inhibitor, JTR005, were then assessed at equimolar concentrations to demonstrate the differential capacities of these two agents to limit Aβ secretion from SH-SY5Y cells (Figure 3C). JTR-009 consistently inhibited secreted Aβ at concentrations as low as 10 nM (Figure 3C). The fifth inhibitor in the series, JTR-005, was a typical comparative control compound since it is also a tricyclic planar compound but without a benzimidazole backbone. JTR-005 also targeted the APP 5′UTR (>PrP 5′UTR), although at 10-fold less potency than JTR-009. Consistent with this fact, JTR-009 reduced Aβ levels at lower concentrations than JTR-005 without causing any significant cell death as measured by an LDH cytotoxicity assay at concentrations up to 10 µM (Figure 3C).

The relative cellular toxicity of the APP 5′UTR inhibitors JTR-005 and JTR-009 was determined by the MTT assay for cellular mitochondrial activity. Figure 3D shows a representative experiment where the mean values for MTS absorbance was a reflection of viability after treatment of the cells with JTR-009 compared to JTR-005 at 0.01 µM (Percent of maximal viability for each treatment ± SEM (N = 3)). These results consistently showed that mitochondrial staining was compromised by increased doses of JTR-005 whereas JTR-009 sustained cellular viability at 80% compared to controls, for concentrations of the drug as high as 100 µM. In sum, JTR-009 increased the relative viability of SH-SY5Y from escalating doses from 1 nM to 1 µM and sustained high viability to 30 µM concentrations. We thus ranked JTR-009 as our most potent and least toxic APP 5′UTR inhibitor (Figure 3).

### C. JTR-009 as a low-dose acting compound in both SH-SY5Y cells and primary cortical neurons relative to posiphen as a well-tolerated APP 5′UTR acting agent

**Figure 4:**
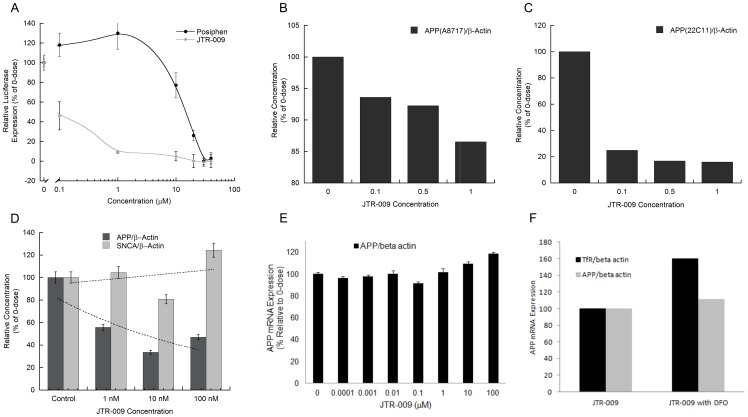
Evaluation of the potency and selectivity of APP blocker-9. **Panel A**: Dose responsive measurement of the capacity of JTR-009 to limit APP 5′UTR-luciferase expression relative to posiphen, a known APP translation blocker (JTR-009: IC_50_ = 0.1 µM; posiphen: IC_50_ = 5 µM, N = 4). **Panel B**: Dose-responsive reduction APP levels in SH-SY5Y cells treated 48 hours at 0.1 µM, 0.5 µM and 1 µM JTR-009. Western blot for APP levels using N- terminal 22C11 antibody (standardization with β-actin as loading control). Bottom Panel: Histogram quantitation of the relative expression of APP/β-actin in SH-SY5Y cells. **Panel C**: Lysates from the experiment in Panel B was analyzed by Western blotting using APP the C-terminal specific (A8717) antibody and β-actin antibody. Bottom Panel: histogram quantitation of the relative expression of APP/β-actin in SH-SY5Y cells from autoradiographic film subjected to densitometry (N = 3). **Panel D**: Dose-responsive capacity of JTR-009 to limit APP expression in primary E-18 mouse neurons (1 nM). The relative α-synuclein (*SNCA*) expression was calculated. Shown, the combined data was graphed into a histogram where mean values from separate assays were calculated from densitometry of Western blots (N = 5). **Panel E**: Real-time qPCR measurement of the dose-responsive measurement of the levels of APP mRNA in SH-SY5Y cells treated with escalating concentrations of JTR-009 for 48 hours. **Panel F**: Equivalent real-time qRT-PCR analysis to measure APP mRNA and TfR mRNA levels in SH-SY5Y cells after 48 h treatment with 25 µM desferrioxamine (DFO) (Positive control for qRT-PCR analysis shown in Panel E).

We previously characterized the anticholinesterase, phenserine (PS), and its (+)-enantiomer, posiphen, as APP 5′UTR-directed drugs. Indeed, posiphen passed Phase 1 clinical trials for AD, exhibiting anti-amyloid efficacy [31], [32], [33], [34]. A direct comparison of the inhibitory potency of JTR-009 and posiphen is shown in Figure 4 (Panel A). Here, the comparative IC_50_ of posiphen to reduce APP 5′UTR-luciferase expression was 5 µM whereas JTR-009 was maximally 50-fold more potent (see also Table 2). At 0.1 µM drug concentrations, JTR-009 treatment reduced APP 5′UTR activity two-fold (N = 4, p<00015) whereas posiphen increased APP 5′UTR activity by 15% (p<0.0299 under matched conditions). These experiments were highly reproducible and confirmed the potency of the action of JTR-009 compared to posiphen as a well-tolerated APP 5′UTR-directed translation blocker that had previously been reported to display anti-amyloid efficacy *in vivo* [33] [34].

**Table 2:** Comparative IC_50_ of JTR-009 relative to posiphen to inhibit APP 5′UTR driven luciferase expression relative to suppression of APP and Aβ levels in SH-SY5Y cells and primary neurons.

Drug	APP 5′UTR Inhibition (IC_50_)	APP and amyloid-beta Inhibition (IC_50_)	Specificity (×10 of IC_50_)	Toxicity (MTT)
JTR-009	100 nM	100 nM(max 10 nM)	β-actin (×20), αsyn (×20)	100 µM
Posiphen	5 µM	1 µM (Refs 27, 33)	β-actin (×20),αsyn (×1)	100 µM

In the experiments represented by Panels B and C of Figure 4, at 80% confluence, SH-SY5Y cells were tested with JTR-009 at the 0.1 µM, 0.5 µM and 1 µM concentrations indicated. After 48 hours of treatment, the cells were collected in lysis buffer and analyzed by multiple western blots. We consistently observed a low dose efficacy of JTR-009 to limit APP expression in SH-SY5Y cells whereas, even at higher doses, the compound maintained cell viability (N = 7). These western blot data demonstrated that JTR-009 consistently reduced APP levels (β-actin standardized in SH-SY5Y neural cells) (Figures 4B,C) at equivalent concentrations. Here, both A8717 (APP C-terminal specific in Panel C) and 22C11 (APP N-terminal specific in Panel B) antibodies were used to detect APP whereas β-actin was used as a loading standard in two separate experiments. In sum, JTR-009 effectively limited APP production on SH-SY5Y cells at doses as low as 100 nM.

Shown in Figure 4D, JTR-009 reduced APP levels by 60% at concentrations as low as 10 nM in primary mouse cortical neurons while α-synuclein (*SNCA*) levels were unchanged and cell viability was maintained. The histogram shows the measured levels of APP as assessed with the 22C11 APP specific N- terminal antibody for western blots after standardization with β-actin. The average pair-wise reduction of *SNCA* levels after control/JTR-009-treatment was at a 50% threshold for 0.001 µM drug exposure to cells for 48 hours. These same treatment conditions left α-synuclein expression unchanged (p<0.01, analyzed by ANOVA).

Of significance, APP blocker-9 did not reduce APP mRNA levels to account for the reduction of precursor protein as judged by qRT-PCR analysis (N = 4). In fact, APP mRNA levels were unchanged at increasing doses from 0.1 nM to 10 µM drug (Figure 4E). Thus, at concentrations that ablated APP protein expression by >75%, APP mRNA levels were unchanged. Indeed the steady state levels of APP mRNA were found increased at concentrations of JTR-009 that were greater than 10 µM (48 h treatment). Specifically, exposure of SH-SY5Y cells to 100 µM JTR-009 increased APP mRNA levels by ∼10% whereas APP protein expression was nearly completely blocked (Figure 4 Panels A–D compared to Panel E). These data underscore that JTR-009 blocks APP expression at the level of APP mRNA translation and not at the level of APP transcription.

As a positive control, transferrin receptor mRNA levels were 2-fold increased in the presence of 48 h iron chelation with desferrioxamine (Fig. 4F). By contrast, APP mRNA was unchanged by iron chelation with desferrioxamine, as we previously reported [6].

### D. Mechanism of action: JTR-009 is a benzimidazole and irreversibly replaces IRP1 from binding to the APP 5utranslated region

**Figure 5:**
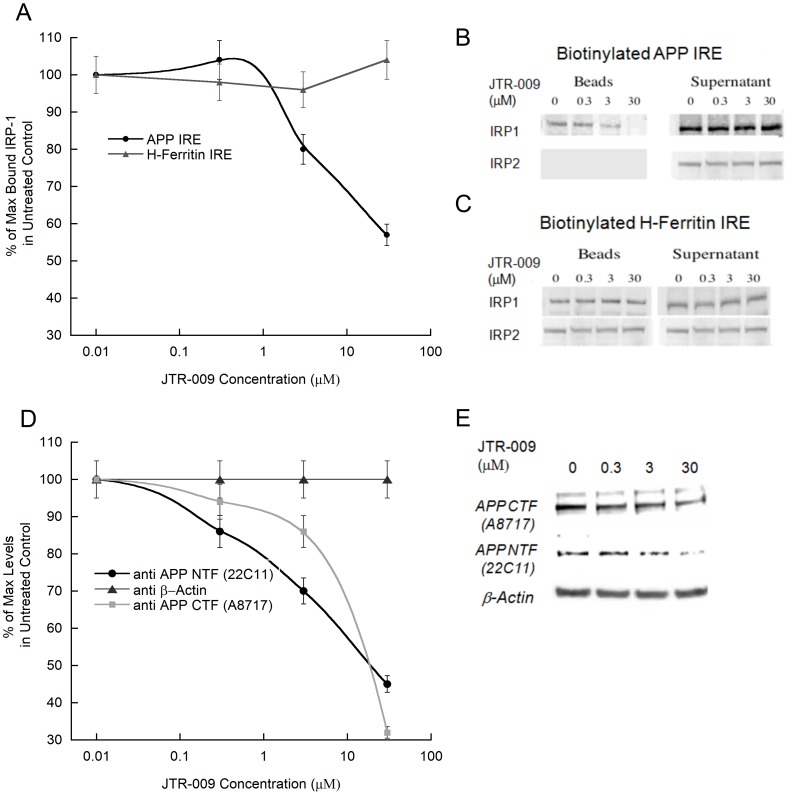
RNA pulldown assay to measure the dose-dependent capacity of the cyclic benzimidazole JTR-009 to substitute for IRP1 binding to APP 5′UTR sequences in SH-SY5Y cells: correlated repression of APP translation. RNA pulldown assays were conducted as ilustrated in Figure 6 and as described by Cho et al., 2010 [6] **Panel A and B**: Representative RNA binding assays in which recovered beads measured the dose-responsive capacity of JTR-009 (0 µM 0.3 µM, 3 µM and 30 µM) to inhibit IRP1 binding to 30 base biotinylated probes encoding the APP 5′UTR. In Panel B Western blots measured relative levels of IRP1 and IRP2 bound to biotinylated RNA probes for APP IRE sequences after recovery in steptavidin bead fractions. Densitomteric quantitation of bead-specific IRP1 is shown in Panel A. **Panel C**: Measurement of the dose-dependent off-target action of JTR-009 to suppress H-ferritin IRE binding to SH-SY5Y specific IRP1 and IRP2 (bead fraction). **Panel D and E**: Dose-dependent decrease of APP levels in response to JTR-009 measured in the supernatants of bead fractions (experimental<control set (p<0.00 1). **Panel E**: Western blots of lysate supernatants showing APP as measured using the N terminal specific 22C11 and C-terminal specific A8717 anitibodies. **Panel D**: Densitometric quantitation of the data in Panel E to measure the extent to which JTR-009 dose dependently repressed APP expression in SH-SY5Y cells (Dunnetts test, p = 0.03). Data from 5 separate trials, each in triplicate.

**Figure 6:**
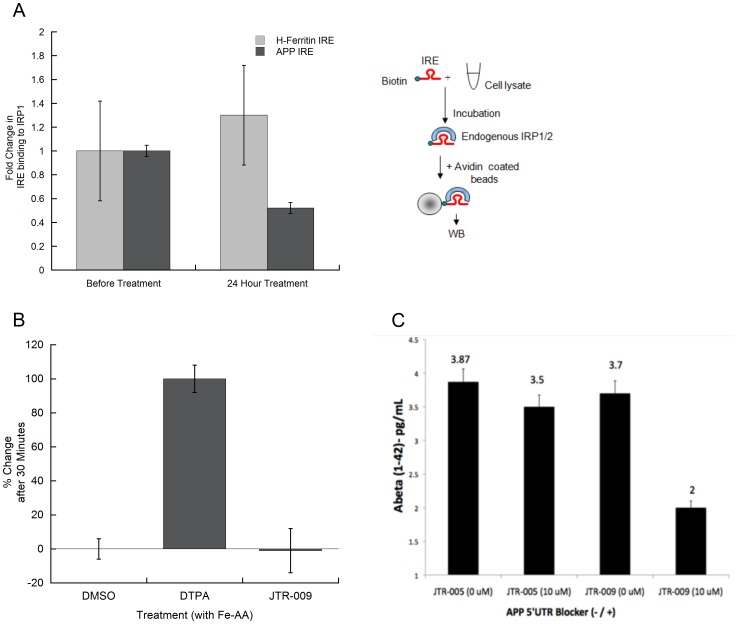
RNA binding assay to measure the capacity of JTR-009 to replace IRP1 binding to biotinylated probes encoding core APP IRE sequences compared to IRP1 binding to the H-ferritin IRE sequences (N = 4). **Panel A**: Top Panel: Cartoon representation of the protocol employed to detect RNA binding between IRE probes and IRP1 in SH-SY5Y cell lysates. Bottom Panel: Effect of JTR-009 treatment of SH-SY5Y cells (24 h, 10 µM) to alter ferritin-H IRE binding to IRP1 compared to that of the APP IRE. **Panel B**: The calcein assay for iron levels in SH-SY5Y cells in response to treatment with JTR-009. Cells were treated with either DMSO (negative control), extracellular iron chelator (DPTA), or JTR-009 at 10 µM for 48 hours. **Panel C**: The anti-amyloid-Aβ-42 efficacy of the APP 5′UTR inhibitors JTR-005 and JTR-009, as measured in conditioned medium from SH-SY5Y neuronal cells (Chemiluminescent BetaMark x-42 ELISA assay from Covance, inc). Data shows the mean values for the reduction of levels of Aβ-42 ± SEM (N = 3) after 72 h treatment of the cells with JTR-009 compared to JTR-005 at 10 µM (p<0.01), analyzed by ANOVA.

When evaluating the mechanism of JTR-009, we noted that a low molecular weight RNA intercalator had been previously reported from the same molecular library source [35], and this agent had been shown to prevent a tau-mRNA splicing event that can cause frontotemporal dementia [35]. Therefore, using SH-SY5Y cells, we employed biotinylated RNA pulldown assays to measure the effect of JTR-009 on the binding of IRP1 to the APP 5UTR (Figures 5 and 6). Multiple biotinylated RNA pulldown assays provided data to confirm that this benzimidazole-based molecule acted “on-target” to substitute for IRP1 interaction as a repressor of APP translation at the site of the APP iron-responsive element RNA stem loop. In Panel B of Figure 5 we showed that administration of of JTR-009 to SH-SY5Y cells dose dependently diminished the percent of IRP1 bound to biotinylated APP IRE RNA probes. Densitometry, as shown in Panel B, quantitated that IRP1 binding was reduced by 20% (±2%) at 0.3 µM, by 50% (+/−5%) at 3 µM, and was completely inhibited at 30 µM (±1%) JTR-009 (N = 7, p = 0.003). Confirming specificity, full interaction between IRP1 and IRP2 and the H-ferritin for IRE probes were always maintained during conditions of induction with JTR-009 (Densitometry in Panel A is shown to reflect a representative Western blot in Panel C)).

The data in Figure 5E provides a representative Western blot experiment from 7 independent experiments when using JTR-009 at 0.3 µM, 3 µM and 30 µM concentrations to inhibit APP expression in the lysate supernatant fractions of SH-SY5Y cells subjected to RNA pulldown analysis (Figure 6A). Consistently, we observed that JTR-009 blocked APP expression as shown by the decreased levels of the precursor when detected with both C- and N-terminal specific antibodies. This reduction of APP levels directly correlated to the elimination of IRP1 binding to APP 5′UTR sequences. Figure 5D shows densitometry to obtain the average reductions of APP levels from multiple Western blots as represented by Panel E (N = 6 for each set). We observed a 70%+/−5% reduction of APP at 30 µM (N = 4, p = 0.02) and 35% at 3 µM JTR-009 (p = 0.01, Dunnetts post-hoc test).

### E. JTR-009 reduced APP expression via its IRE in an iron independent manner

**Figure 7:**
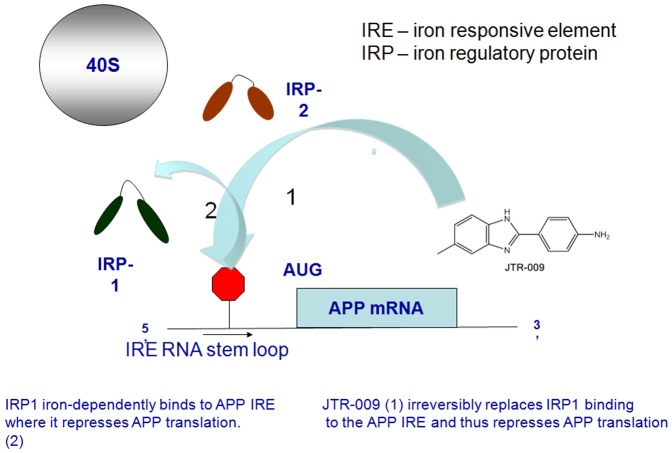
Model for the action of the bezimidazole JTR-009 to act as an inhibitor of APP translation by irreversibly replacing the iron dependent translation repressor IRP1 from interacting with APP 5′UTR sequences. Binding of JTR-009 selectively targeted APP 5′UTR sequences and then was found to repress APP levels leading to reduced amyloid levels without perturbing cellular iron uptake.

To confirm an “on-target” mechanism for JTR-009 and its relative iron-independence when acting via APP 5′UTR, we had previously performed a molecular determination of the iron-dependent, reversible binding of IRP1 to the APP IRE stem loop (K_d_ = 30 pM) [6]. Our data shown in Figure 5 is consistent with the model that JTR-009 substituted for IRP1 binding to APP 5′UTR sequences. In Figure 6A, we compared the extent to which JTR-009 decreased IRP1 binding of APP-IRE in SH-SY5Y cells compared to H-Ferritin-IRE RNA probes. Here, the results consistently showed that 3 µM JTR-009 (48 h exposure) reduced IRP1 binding to the APP IRE by 2-fold whereas under the same conditions this benzimidazole displayed no inhibitory change in binding to H-ferritin IRE probes. These data were consistent with the conclusion that JTR-009 bound selectively to the APP IRE sequences and not to related RNA probes encoding the ferritin-H IRE, an observation consistent with the proposed mechanism of action of JTR-009 as shown in Figure 7.

We directly measured the effect of JTR-009 on iron homeostasis in a calcein uptake assay in SH-SY5Y cells (Figure 6B). The drug (DTPA) was the positive control as an extracellular chelator that completely blocks iron uptake. Normalized results were graphed and these data showed that DMSO caused 0±6% inhibition of the amount of calcein stain (proportional to iron levels) and DTPA caused 100±8% inhibition. Of note, JTR-009 induced a −1±13% inhibition of calcein staining. Thus under these conditions, we systematically observed that JTR-009 had little or no effect on iron uptake (TfR dependent and independent uptake pathways).

Aß-42 peptide is the critical APP derived peptide to trigger the aggregation of amyloid in both AD and DS and is critically linked to tau induced neurotoxicity [36]. Thus, we tested and reproducibly demonstrated that JTR-009 limited Aβ-42 secretion from SH-SY5Y cells by more than two fold. In this representative experiment, a chemiluminescent BetaMark x-42 ELISA assay for Aβ-42 measurement was employed (Covance, inc). The assays were carried out according to the manufactures conditions such that the standard curve was linear and the measured points were within the ‘standards’ range. JTR-005 exhibited only a 20% reduction of Aβ-42 output after the same 3-day treatment as that of JTR-009 (72 hour treatment).

In sum, these experiments demonstrated that JTR-009 operated by direct pathways to reduce APP translation an Aβ-42 output and the compound did not act via indirect pathways as a secondary iron chelator in which case the drug would be expected to activate binding of IRP1 as a translational repressor. Our working model shown in Figure 7 evidenced that JTR-009 interacts directly with the APP IRE RNA stem loop.

## Discussion

RNA-directed drugs have long been used to treat infectious diseases, e.g. antibiotic aminoglycosides, and small RNA-directed molecules have been used to control gene expression in cell culture models [37] (e.g. therapeutic control of viral Hepatitis C/HIV gene expression [38]). In mammals, endogenous up-regulation of the translation of the iron storage protein ferritin by ‘yohimbine’, as a ligand of its native RNA, was shown to enhance protection of cells from Fe-catalyzed oxidative stress [39].

We previously demonstrated that it is feasible to generate pharmacological anti-amyloid efficacy by targeting the 5′UTR sequences in the amyloid precursor protein (APP) transcript [40]. Certainly, iron chelation with desferioxamine increased RNA protein interactions between Iron-regulatory Protein-1 and the transcript that translates APP, a pharmacological treatment predicted to reduce levels of Aβ peptide both *in vitro* and *in vivo* [6], [8]. We also reported on the use of FDA pre-approved drugs as inhibitors directed against the 5′UTR of the APP transcript. For example, the tricyclic benzoxazole paroxetine (serotonin-specific reuptake inhibitor) and the antioxidant N-acetyl cysteine (NAC) suppressed APP 5′UTR driven translation of a luciferase reporter gene and actively limited APP mRNA translation and Aβ production in neural cells lines without altering amyloid precursor-like protein-1 (APLP1) levels [41]. Both paroxetine and NAC subsequently displayed *in vivo* anti-amyloid efficacy in APP transgenic mice [8], an approach we extended to the use of agents of potential benefit to Parkinson's disease patients for whom proven FDA drugs limited the translation of *SNCA* mRNA by targeting *SNCA* 5′UTR sequences [32].

As a second example of the use of APP 5′UTR-directed inhibitors, the well-characterized drug posiphen, was found to block translation of APP and limit amyloid levels, both in neural cells lines [42], [43] and in mice *in vivo* [33]. Posiphen operated in the micromolar range and, like the other well-characterized APP 5′UTR-directed inhibitor pifithrin, limited expression of APP but also that of β-actin and α-synuclein (Figure 3).

In this report, we introduced the mechanism-of-action of a new class of APP translation inhibitors with improved potency and selectivity to the uniquely folded APP 5′UTR target. We characterized the *in vitro and ex vivo* action of APP blocker-9 (JTR-009), a significantly more potent and more selective translation blocker than posiphen. The data in Table 2 summarizes the effectiveness of JTR-009, relative to the well-tolerated anti-APP 5′UTR inhibitor posiphen, to repress APP expression in SH-SY5Y cells and in primary neurons.

Our stringent counter-screens identified that the ninth of the series of the thirteen APP translation blockers displayed a 2-fold higher capacity to inhibit APP 5′UTR-conferred luciferase expression in pIRES-APP-5′UTR transfectants relative to PrP 5′UTR repression of luciferase expression in the pIRES-PrP-5UTR cells. Other APP 5′UTR inhibitors such as JTR-008 (a benzothiazole) did not exhibit selectivity in this counter-screen (Table 1). In sum, APP 5′UTR directed translation blockers, such as JTR-009, offer an improvement on the use of both phenserine and its stereoisomer posiphen, both of which were shown to block APP translation and limit Aβ expression upon administration to human clinical trial volunteers [34].

Contrary to the action of iron specific chelators that promote repression of APP translation by IRP1, the mechanism-of-action of JTR-009 in SH-SY5Y cells was consistent with this small molecule acting “on-target” to directly intercalate into the RNA stem loop that encodes the iron-responsive element RNA stem-loop in APP mRNA. The data in Figure 5 was representative of multiple tests (N = 8), showing that JTR-009 reduced APP mRNA translation in SH-SY5Y cells correlated with its substitution for IRP1 binding to APP 5′UTR sequences. Consistent with this, several of the chemical features of the hits from the second HTS undertaken at Columbia University screen were common to the first screen, including a potential structure-activity relationship (SAR), since five of our thirteen leads fell into two related classes of compounds: compounds with benzimidazole backbones and compounds with benothiazole backbones. These compounds may also intercalate into the APP 5′UTR RNA secondary structure as aromatic planar molecules, each capable of forming hydrogen bonds with the phosphate backbone of RNA helix (26).

Two models can be tested to explain how JTR-009 acts to inhibit APP translation with such high selectivity. JTR-009 is a benzimidazole that may alter intracellular kinase signaling so as to replace IRP1 for another, as yet unidentified, translation repressor of APP via its 5′UTR. However, a more likely mode is that JTR-009 operates by a mechanism of drug action similar to that observed for other benzimidazoles [26], [44] and that is, by selectively intercalating between the bases stacked in the unique RNA stem loop folded by APP IRE sequences (see Figures 1A and Figure 7). This model is further backed by examples of other RNA-targeting drugs that intercalate with the tau mRNA stem loop that can modulates splicing events relevant to the onset of frontotemporal dementia [45]. By irreversibly replacing IRP1 as the binding partner of the APP IRE stem loop, JTR-009 could directly interfere with ribosome scanning of the APP 5′UTR by the 40S ribosome prior to translation of the precursor (see model in Figure 7). This tricyclic benzimidazole compound did not change the binding pattern between IRP1 and the classic H-ferritin IRE (Figure 6), confirming its selectivity [6]. The IC_50_ for its translational inhibition of APP was <1 µM, as established from dose-responsive assays [46].

We used calscein assays to determine that pharmacological reduction of APP was not mediated by indirect changes to iron levels to change APP expression and Aβ production (Figure 6B). Therefore, JTR-009 was sufficiently selective to the APP IRE stem loop to reduce APP, while not changing several biomarkers of intracellular iron status of cells. Since APP translation was previously shown to be closely controlled in response to intracellular iron levels, this scenario was originally thought to be a possible mechanism [6]. Instead, the binding of IRP1 and IRP2 to the ferritin IRE was unaffected by exposure of neural cells lines to JTR-009 (Figures 5 and 6). Together with the lack of changes of cellular calcein stains, these data evidenced that JTR-009 exerted a direct action on APP and blocked amyloid without gross perturbations to iron homeostasis (See Figure 7).

The prolonged use of JTR-009 would be predicted to limit APP mRNA translation to thus reduce intracellular APP levels, a situation that causes greater iron retention within cells with the loss of APP facilitated iron export by ferroportin [5]. However, our results showed little or no change in intracellular iron content after treatment of SH-SY5Y (or primary neurons) with JTR-009. Thus, the reduced amount of iron to be exported predicted from loss of APP could have been compensated by less iron uptake by transferrin bound or unbound pathways [5]. Another attractive model is that JTR-009 increased translation of ferroportin at the same time as it inhibited APP production, which explains the reason that intracellular iron levels were unchanged in response to JTR-009. For example, JTR-009 could have altered IRP1 binding to the IRE in DMT1 mRNA and/or ferroportin mRNA to account for the predicted compensatory increase in cellular iron expected by loss of APP expression since APP is an iron export ferroxidase [5].

Consistent with the capacity of JTR-009 to maintain correct iron balance and to operate as a potent anti-amyloid agent, we observed this benzimidazole-enhanced cell viability by use of MTS and LDH assays. In fact, JTR0-009 was pharmacologically non-toxic in SH-SY5Y cells as measured by MTS assay (Figure 3D). Dose-responsive measurements confirmed the IC_50_ against the APP 5′UTR to be in the 1 nM to 100 nM range while the APP 5′UTR-directed JTR-009, only displayed cell toxiciy at concentrations >30 µM in SH-SY5Y cells and in primary neurons. Significantly, JTR-009 exhibited a similar toxicity at 100 µM in SH-SY5Y cells as was previously demonstrated for posiphen (+ve control) [21].

Certainly JTR-009 was a highly selective APP inhibitor that operated at very low concentrations in the nanomolar range, as has been evident in other kinase inhibitors. The series of experiments shown in Figure 4 revealed that low doses of JTR-009 typically reduced APP expression. Intracellular APP inhibition was quantified by densitometry such that both the N-terminus 22C11 antibody and the C-terminus A8717 antibody of APP were cross-referenced. We found that each of the APP 5′UTR inhibitors lowered Aβ secretion from SH-SY5Y neuroblastoma cells (Figure 2), albeit at levels near the limits of detection for Aβ (pg/ml range).

Of note, the potency of JTR-009 to inhibit APP 5′UTR conferred translation was greater than posiphen (Figure 4A). In primary mouse E-18 neurons, JTR-009 inhibited APP levels with no reduction of β-actin at doses as low as 1 nM and, dose-responsively, as high as 100 nM (Figure 4D). The same concentrations of JTR-009 demonstrated no significant changes to α-synuclein levels (the western blots were standardized to β-actin) (Figure 4D).

We sought to more stringently identify novel agents as translation blockers specifically of APP 5′UTR sequences by counter-screening APP inhibitor leads against the PrP (Vt-2) 5′UTR. To this purpose, we employed SH-SH5Y cells stably transfected with the pIRES-APP-5′UTR and pIRES-PrP-5′UTR constructs, which expressed a luciferase reporter driven respectively by the APP and PrP (V2) 5′UTRs (Figure 1D). While undertaking the counter-screens, we identified an unexpectedly close 56% identity between PrP and APP mRNAs as well as a putative IRP1 binding site in the 5′untranslated regions in both mRNAs. These similarities provided an explanation as to why our dose-responsive counter-screen identified similar IC_50_ values for both APP and PrP 5′UTR dependent inhibition of luciferase reporter expression (Table 1).

Consistent with these observations, recent reports linked the function of both APP [5], [6] and PrP to a common role in iron transport where both these neurodegenerative gene products are differentially increased by iron [29], [47]. APP mRNA encodes a fully functional IRE [6] and our bioinformatic alignments showed that secondary structures of the 5′UTRs of several neurodegenerative transcripts also appear to encode IRE RNA stem loops, for instance in the related 5′UTR of PrP mRNAs (Figure 1). This unexpected close sequence similarity between the PrP 5′UTR to that of the APP 5′UTR may explain why many of the top APP specific inhibitors were found to also repressed prion (PrP) expression. Certainly these two neurodegenerative proteins are linked with a common role in iron transport and metabolism [29] where recent discoveries also show an *in vivo* interaction of the Aβ amyloid protein and the PrP prion during the progression of neurodegenerative disease [48]. However, these findings also underscored the specificity of JTR-009, which inhibited APP expression 10-fold more actively than that of the prion PrP protein.

## Conclusions

The single major conclusion from this work is that APP 5′UTR sequences indeed are a significant regulator of Aβ precursor expression in neural cells. In this report, it was via APP 5′UTR-dependent pathways that we pharmacologically limited the steady state levels of APP not only in SH-SY5Y cells but also in primary cortical neurons. These APP 5′UTRs-directed inhibitors are currently being tested as representatives of a class of intercalators that exhibit high selectivity in reducing APP production in SH-SY5Y neuroblastoma cells. They also limited Aβ production with little or no perturbation of cellular iron homeostasis while maintaining neuronal viability. Such capabilities are the requirements for future drugs with therapeutic potential for AD and Down syndrome. On a technical level, the optimal compound was shown to be a benzimidazole, JTR-009, that limits APP translation at less than one nanomolar concentrations with little evidence of neurotoxicity. This tricyclic compound inhibits APP translation by directly interacting with the IRE in the 5′UTR of APP mRNA, irreversibly replacing IRP1 as a repressor of translation. JTR-009 was 10-fold more effective to limit APP translation than posiphen, a well tolerated APP 5′UTR-directed translation blocker [33].
